# Recurrent contracted sockets treated with personalized, three-dimensionally printed conformers and buccal grafts

**DOI:** 10.1177/11206721211000013

**Published:** 2021-03-11

**Authors:** ALW Groot, Jelmer S Remmers, Roel JHM Kloos, Peerooz Saeed, Dyonne T Hartong

**Affiliations:** Amsterdam UMC, Department of Ophthalmology, Amsterdam Orbital Center, University of Amsterdam, Meibergdreef 9, Amsterdam, the Netherlands

**Keywords:** Anophthalmic socket, orbital disease, orbital surgery, orbital trauma, eyelid disease: eyelid reconstruction, oculoplastic eyelid/lacrimal disease, immune disease of conjunctiva, cornea/external disease

## Abstract

**Purpose:**

Recurrent contracted sockets are complex situations where previous surgeries have failed, disabling the wear of an ocular prosthesis. A combined method of surgery and long-term fixation using custom-made, three-dimensional (3D) printed conformers is evaluated.

**Methods:**

Retrospective case series of nine patients with recurrent excessive socket contraction and inability to wear a prosthesis, caused by chemical burns (*n* = 3), fireworks (*n* = 3), trauma (*n* = 2) and enucleation and radiotherapy at childhood due to optic nerve glioma (*n* = 1) with three average previous socket surgeries (range 2–6). Treatment consisted of a buccal mucosal graft and personalized 3D-printed conformer designed to be fixated to the periosteum and tarsal plates for minimal 2 months. Primary outcome was the retention of an ocular prosthesis. Secondary outcome was the need for additional surgeries.

**Results:**

Outcomes were measured at final follow-up between 7 and 36 months postoperatively (mean 20 months). Eight cases were able to wear an ocular prosthesis after 2 months. Three cases initially treated for only the upper or only the lower fornix needed subsequent surgery for the opposite fornix for functional reasons. Two cases had later surgery for cosmetic improvement of upper eyelid position. Despite pre-existing lid abnormalities (scar, entropion, lash deficiency), cosmetic outcome was judged highly acceptable in six cases because of symmetric contour and volume, and reasonably acceptable in the remaining two.

**Conclusions:**

Buccal mucosal transplant fixated with a personalized 3D-designed conformer enables retention of a well-fitted ocular prosthesis in previously failed socket surgeries. Initial treatment of both upper and lower fornices is recommended to avoid subsequent surgeries for functional reasons.

## Introduction

The goal after ocular evisceration or enucleation is to fit an ocular prosthesis that resembles a natural eye as closely as possible. In order to fit an ocular prosthesis, deep eyelid fornices with sufficient conjunctival lining are mandatory. Shortness of conjunctival lining with shallow or absent fornices can occur in various conditions, including previous external beam radiation, multiple previous extrusions of an orbital implant, immunologic diseases such as mucous membrane pemphigoid or Stevens Johnson syndrome, or after chemical or thermal trauma. Standard procedures for fornix reconstruction include fornix-deepening sutures with or without the use of buccal mucosal transplant.^1^ A dermis fat graft (DFG) may also be used to increase the lining of the socket. In a subset of patients these standard procedures unfortunately fail, and severe recurrent contraction of the socket results in the inability to wear any ocular prosthesis. This situation is referred to as stage 5 contracted socket as defined by Krishna^2^, where “there is recurrence of contraction of the socket after repeated trial of reconstruction”.

One of the possible underlying problems for inadequate wound healing and contraction is ischemia with insufficient vascularization. In the wound healing process, myofibroblasts play a role as modulator in granulation formation and tissue contraction.^3,4^ Mucosal tissue is prone to contraction, but this may be reduced by mechanical restraint.^5^ Accordingly, we hypothesized that two main problems have to be overcome: to let the graft attach to a vital surface, and to prevent the graft from contracting. Therefore, the mucosal transplant has to be both attached to a fresh wound bed and fixated in the correct position for a long time.

As previously described by this group, a customized three-dimensionally (3D) printed conformer can be designed to have the optimal dimensions to fit in the operated socket and to serve as a pressure device for the transplanted mucosa.^6^ Standard, non-custom-made conformers are available and might be a reasonable alternative. 3D design offers the advantage to modify the conformer, by adding fixation holes for fornix-deepening sutures and a central wing-shaped horizontal extension (a “lip”) for fixation to the superior and inferior tarsal plates. This will enable firm fixation and avoids luxation. Another advantage is that the anterior part of the conformer (generally defined by the position of an eye within the orbit) can be mirrored from the healthy eye, enabling a well-formed contour that will be translated to the socket during the 2 months fixation. After removal, this conformer contour can directly be translated to the definite prosthesis. In this study we describe our experience with nine patients were this method of personalized 3D-printed conformer with long-term mucosal transplant fixation was applied.

## Methods

### Patients

This retrospective case series includes nine patients who were unable to hold an ocular prosthesis due to a recurrent contracted socket after previously failed surgeries, and who were thus graded as being Krishna stage 5. Patients came to our tertiary referral center at the Amsterdam University Medical Center between January 2016 and October 2019. Patient demographics are shown in Table 1. Three patients had a history of a chemical burn all with alkali agents, three had fireworks trauma, two had a history of trauma and multiple surgeries, and one patient had had an enucleation at childhood followed by postoperative radiotherapy due to an optic nerve glioma. Median age at initial trauma or initial surgery was 14 years (range 2.5–51 years) and the average amount of previous surgeries involving the fornix was three (range 2–6). Average age at socket reconstruction was 44 years. The medical ethical committee of the Amsterdam Medical Center approved the study and the study adhered to the ethical principles outlined in the Declaration of Helsinki as amended in 2008. The patients gave written consent for the use of their anonymous information and the two patients whom it regards separately approved the presentation of pre- and post-operative pictures shown in this article.

**Table 1.** Patient characteristics.

Case	Gender	Cause	Age at initial surgery	Previous fornix surgeries or procedures	Krishna stadium*	Age at socket reconstruction	Follow up (months)
1	F	Chemical (alkali)	2.5	Symblepharolysis	5	35	31
				Fornix reconstruction with adhaesiolysis and amnion membrane transplant			
				Symblepharolysis and amnion membrane transplant			
				Fornix reconstruction with oral mucosal graft			
				Dermis fat graft with fornix deepening procedure			
				Conformer with temporary tarsoraphy			
2	M	Fireworks	12	Pentagon excision upper eyelid, oral mucosal graft, tarsoraphy	5	27	32
				Symblepharolysis, fornix reconstruction with amnion membrane transplant			
				Entropion correction upper eyelid			
				Enucleation + dermis fat graft with conformer			
3	F	Trauma	10	Socket reconstruction with dermis fat graft	5	45	27
				Possibly more procedures, unsure about medical history			
4	M	Chemical (alkali)	51	Debridement of necrosis, amnion membrane transplant	5	52	15
				Additional amnion membrane transplant in fornices			
				Evisceration after corneal perforation, symblepharolysis			
5	M	Fireworks	26	Symblepharolysis, oral mucosal graft and upper and lower entropion correction	5	27	14
				Entropion correction			
				Symblepharolysis, oral mucosal graft and conformer			
6	M	Trauma	Child age	Reconstruction with diverse transplants (both from mouth and from leg)	5	70	7
7	M	Chemical (alkali)	41	Amnion membrane transplant	5	61	11
				Socket reconstruction with fornix deepening sutures, oral mucosal graft, block excision upper eyelid due to scarring			
				Socket reconstruction with inferior fornix repair and fornix deepening sutures			
8	M	Fireworks	14	Four fornix repairing surgeries, unknown what procedures	5	41	9
				Dermis fat graft with symblepharolysis			
				Symblepharolysis			
				Symblepharolysis with Z plasty and tarsal lidsplit with everting sutures			
				Eyelid reconstruction with symblepharolysis and oral mucosal graft			
9	F	Enucleation due to optic glioma and 2 × radiotherapy	4	Socket reconstruction with debulking of the optic glioma and upper fornix reconstruction with symblepharolysis	5	39	6
				Dermis fat graft with oral mucosal graft and conformer			

* Grade 5: Recurrence of contraction of the socket after repeated trials of reconstruction.^2^

### Custom-made three-dimensional (3D) printed conformer

**Figure 1.**
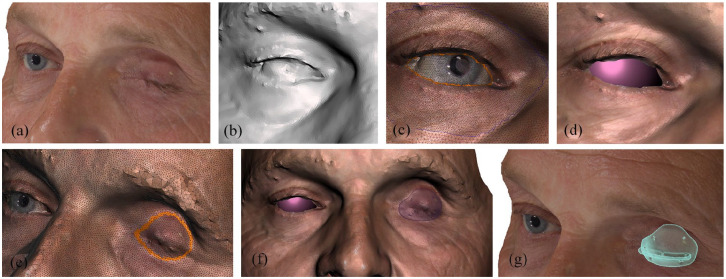
(a) shows the patient preoperatively, (b) the geometry in 3D image of the contralateral, normal eye. The cornea is hard to capture on 3D imaging due to reflections, (c) by mapping the 3D photograph of the patient over the geometry file, it is possible to select the eyelid contour, (d) by selecting this contour, a best fitted sphere is estimated for this contour, which is used as the anterior curvature for the conformer. The thickness is then defined as a standard 2 mm, or thinner/thicker in consultation with the ophthalmologist, (e) the height of the conformer is generally preset at 16-18-20 mm, and checked to fit within the expected bony orbital rims, (f) the conformer is designed within these contours, adding an extension lip and fixation holes, and (g) digital image of the patient with the conformer.

**Figure 2.**
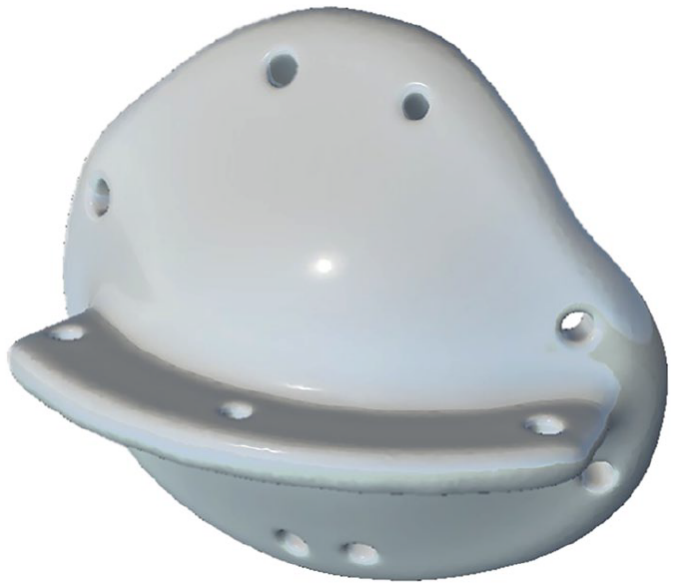
Example of a personalized 3D-printed conformer with horizontal extension “lip” and fixation holes.

The general technique of designing and 3D-printing a conformer was previously described.^6^ In short, at consultation a facial 3D photograph was made, using either the handheld 3D scanner Artec Eva (Artec 3D, Luxembourg) or a static 3D scanning setup (Vectra^®^ M3, Canfield Scientific Europe). The captured data was post-processed using the manufacturers corresponding software (Artec Studio 14 and Vectra Capture module, respectively), exported as a file with .obj extension and loaded into 3D modeling software (Autodesk Meshmixer, Autodesk Inc., San Rafael, USA). The ocularist designed the conformer as an overlay in the 3D software as if it was an adequately sized prosthesis: the anterior curvature defined from the eyelid contour of the fellow eye was used to define the anterior part of the conformer, the posterior part followed this curve (Figure 1). The conformer’s vertical dimensions were determined to fit within the superior and inferior orbital rims, as estimated on the 3D photograph in Figure 1(e). The horizontal dimensions were determined by the available horizontal eyelid width with the addition of 4 mm (medial and lateral fornix space of 2 mm). The horizontal extension lip width was adapted to the eyelid width, and was placed at the lower third of the conformer so that the inferior fornix was half the length of the superior fornix as in the natural anatomy of the eyelids. Finally, fixation holes were created: three at the superior border and three at the inferior border of the conformer, and three at the horizontal extension lip (Figure 2). This conformer was subsequently printed in three different heights (16 mm, 18 mm, and 20 mm), in order to adapt for small variations in available mouth mucosa size encountered during the surgery. We used a stereolithography (SLA) printer (NextDent 5100, NextDent BV, Soesterberg, the Netherlands) with a class IIa long-term biocompatible resin, called “Dental LT Clear resin” (Formlabs, Massachusetts, USA).

### Surgery

**Figure 3.**
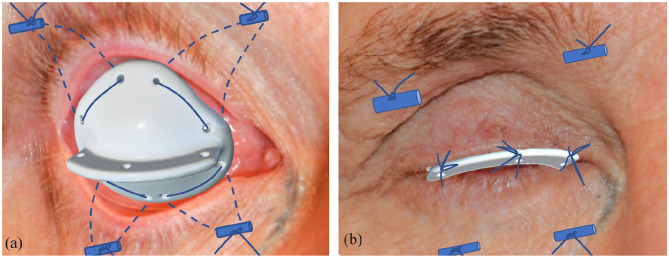
Illustration rendering a conformer in place with: (a) 4× double-armed 6-0 absorbable sutures from the conformer, entering the fornices and extending through the skin where they are fixed to a silicone tube and (b) fornix-deepening as well as tarsal sutures to the horizontal extension lip.

All patients were operated under general anesthesia. Usually cheek, and sometimes lip mucosa was marked for the largest possible graft (preferably about 3 by 5 cm) without interfering with the Stensen’s duct. The mouth mucosa was infiltrated with a solution of xylometozaline 1% or 2% with adrenalin (1:200.000). The mucosa was dissected from the buccinator muscle. If needed, local coagulation was performed. The wound was either left open for spontaneous granulation or closed using interrupted 5-0 absorbable braided polyglactin sutures, submucosal tissue was trimmed from the graft and perforating holes were made to both extend the size of the graft as well as to enable drainage of wound debris. In the cases where there was hardly any mucosal tissue left in the socket, this means the mouth mucosa should widely cover the conformer up to the extension rim. In case there was insufficient mucosa to create deep fornices or cover the conformer, a second graft was taken from the lip or other cheek. The graft was soaked in gentamycin solution. Gloves were changed and clean instruments were used for the socket part: the socket was centrally opened with a horizontal cut from the caruncle to the lateral canthus, and the available conjunctiva was undermined superiorly and inferiorly. Scarred tissues were released until there was a free subconjunctival movement of the instrument into the forniceal space up to the orbital rim. At this moment we determined which size of the conformer should be used: if the eyelids could not be closed easily any disturbing tissue strand was released, or (in case of general sizing problem) the conformer size was exchanged to one with a smaller height. After determining the size, the conformer was placed apart. The prepared mucosal graft was then introduced in the socket, orientating the graft so that the largest dimensions were at the deepest part of the superior and inferior fornices. In order to achieve this, the mucosal graft was often flipped over 45° making a diamond shape. Next, the medial and lateral mucosal flaps (about 5 mm from the edge) were fixed (with double-armed 6-0 absorbable sutures extending through the skin) in the deeper medial and lateral canthal area in order to create about medial and lateral fornices. Subsequently, 6-0 absorbable running sutures were applied to connect the donor buccal mucosa to the recipient socket conjunctiva superiorly and inferiorly. Care was taken to avoid knots in the socket and therefore the sutures were extended through the skin. The large mucosal draping was then pushed in the correct position by gently fitting the conformer and thus creating a “conjunctival” and “palpebral” part of the conjunctiva as in normal anatomy. Double-armed non-absorbable 3-0 monofilament sutures were transferred from the holes in the conformer through the forniceal folds, picking up the periosteum, and extending through the skin where the knot was fixed on a silicone tube (fornix-deepening sutures). Two fornix-deepening sutures were made at the superolateral and superomedial fornix, and two fornix deepening sutures at the inferior fornix. Finally, three central tarsal fixation sutures (non-absorbable 3-0 monofilament) were introduced from the superior tarsal plate, through the central hole in the horizontal extension lip and subsequently through the inferior tarsal plate (tarsorrhaphy sutures). (Figure 3) Postoperative management consisted of 1-week oral broad-spectrum antibiotics, three weeks of tobramycin 0.3%/dexamethasone 0.1% eyedrops and for some cyclosporin 0.05% eyedrops for several months after the surgery to decrease conjunctival inflammation. After 2–3 months the conformer was removed and an ocular prosthesis was fitted.

## Results

Nine patients, aged from 27 to 70 years old, with recurrent contractions after multiple surgeries not able to wear an ocular prosthesis were treated using this personalized socket reconstruction technique. Mean follow up was 20 months (range 7–36 months).

**Figure 4.**
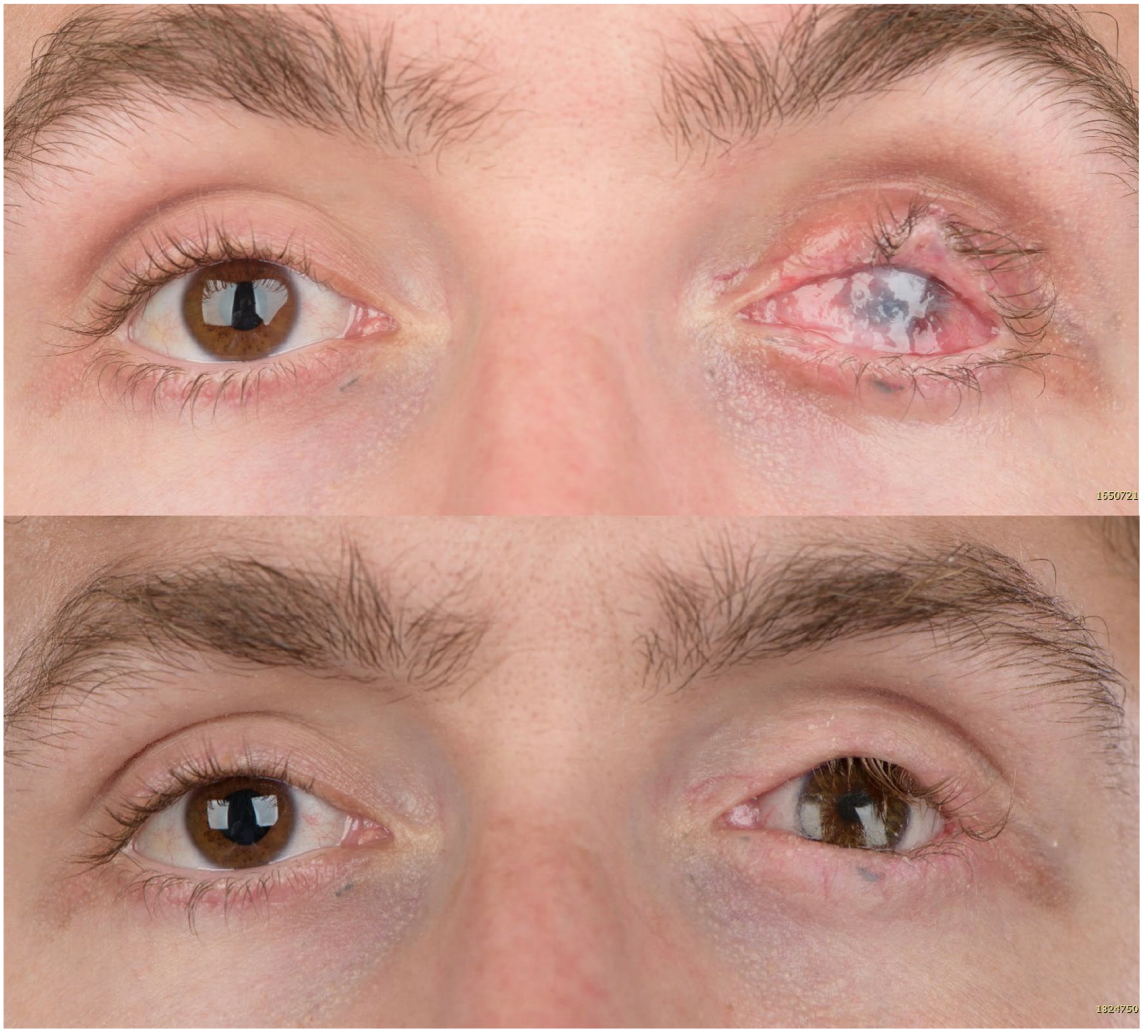
Pre-operative and post-operative photographs of case no 5. Retainment of the conformer is in stable condition. The remaining entropion and superior sulcus volume loss may be treated in a later stage.

In eight out of nine patients sufficient fornices were created, which enabled the retention of an ocular prosthesis (Figure 4). The only exception was the patient who underwent postoperative radiotherapy at a young age. Mean prosthesis dimensions were height 19.7 mm (range 16.5–23.7 mm), width 23.3 mm (16.7–28.7 mm) and thickness 2.3 mm (1.5–3.7 mm) (Table 2).

**Table 2.** Conformer measurements in millimeters.

Case no	Width	Height	Thickness
1	20	17.3	2
2	25	23.7	2.2
3	16.7	16.7	2.3
4	24.6	18.6	2.3
5	26.4	22	2.8
6	28.7	22.9	2
7	22.2	16.5	1.8
8	22.9	21.1	3.7
9	23.3	18.2	1.5

In patients 1, 2 and 3, initially only the inferior fornix was constructed since we judged the superior fornix to be adequate preoperatively. All three patients needed a subsequent reconstruction of the superior fornix since the altered position of the inferior fornix reshaped the socket and prohibited a good fit of the prosthesis. Two patients underwent additional surgery for cosmetic reasons (Table 3).

**Table 3.** Additional surgeries.

Case no	Additional surgery	Motivation	Procedure
1	1	Functional	Entropion correction with buccal mucosa
2	2	Functional	1. Levator desinsertion due to eyelid retraction
			2. Fornix reconstruction with buccal mucosa
3	1	Functional	Entropion correction with buccal mucosa
4	0		
5	1	Cosmetic	Ectropion correction with lateral tarsal strip procedure
6	0		
7	0		
8	1	Cosmetic	Lipofiller upper eyelid due to volume loss
9	0		

All patients already had preoperative loss of levator function and scarred and abnormal position of the lid margin with entropion, misdirected or absent eye lashes, which remained after the socket surgery. For one case, after 23 months of stable socket, it was decided to perform a correction of the superior lid entropion (lid-split with addition of mucosa and a new temporary conformer) resulting in an acceptable situation. The other patients did not want additional entropion surgery. Case 2 had upper eyelid retraction for which we performed a levator disinsertion procedure after 22 months, also with addition of mucosa and again a temporary conformer with good result. Despite the remaining lash abnormalities and lagophthalmos we judged the cosmetic outcome highly acceptable in six out of the eight cases who had symmetric eyelid contour and normal volume or only slight superior sulcus volume deficiency. The cosmetic outcome was reasonably acceptable in one patient with moderate volume loss and pre-existing shortened and irregular horizontal palpebral fissure, and in one patient with pre-existing large volume loss and microblepharon, hence with good contour. All patients were satisfied being able to wear a prosthesis.

Commonly there was a lot of discharge from the socket in the first postoperative weeks, for which regular superficial cleaning was advised. Approximately 2 months after surgery the non-absorbable sutures (4× fornix deepening sutures with silicone bolster, and 3× central tarsorrhaphy sutures) were removed. The conformer was then replaced by a cosmetic prosthesis. In the one case that did not succeed, the conformer luxated from the inferior fornix after 1 month which resulted in obliteration of the lower fornix, and later the superior fornix as well. As a result, a prosthesis could not be fitted.

## Discussion

In this paper we describe a successful method for treating severely recurrent contracted sockets with the use of buccal mucosal transplant in combination with long-term fixation of a personalized 3D-designed and printed conformer. In all cases, standard methods to reconstruct the fornices had failed.

In all eight successful cases, the transplanted mucosa was retained with good vascularization. We experienced that a well-vascularized part of the socket was obtained after the release or excision of the anterior scarred tissue. This vascularized tissue should still be able to serve as a nourishing bed for the free mucosal graft, provided that the graft stays in firm contact with the vascularized bed until the healing process has passed the proliferative phase of wound healing, which takes place between 4 and 21 days after creation of the wound.^7^ In this phase, angiogenesis occurs and extracellular matrix is formed.

To decrease the inflammatory response, we prescribed steroid eyedrops post-operatively. We also empirically started cyclosporin drops simultaneously, as cyclosporin inhibits the release of pro-inflammatory cytokines.^8^ Cyclosporin eye drops are safe and easy to use during a long period. Mild socket contraction seems to benefit from intra-operative 0.02% mitomycin application or four weekly 5FU injections in small comparative studies.^9,10^ As we have not used this, and as our experience is currently limited to these cases, we cannot give clear recommendations on which anti-inflammatory treatment to use for severe contraction.

Looking at the sizes of the postoperative conformers, we were able to preserve the fornices and have thus prevented severe contraction which was seen after their previous surgeries. We assume the long-term fixation of the adequately sized conformer added to this result. In vitro studies have shown the contraction of mucosal tissue to be around 50% after 28 days. Mechanical restraint reduced this contraction to 22%.^11^ Therefore care has to be taken that the fornices are “frozen” in their proper position. Our fixation of the buccal mucosal graft with the custom-made conformer kept the graft attached to the underlying fresh vascular bed, and simultaneously prevented it from mechanical contraction.

Other examples to prevent contraction are the use of a silicone expander and silicone fixative after fornix reconstruction.^12,13^ Other surgeons have used a hard palate composite graft with buccal mucosa. The stiff hard palate is generally less vulnerable to contraction. Choi et al.^14^ described additional fornix surgery in three out of four cases during follow-up due to fornix contraction and Lee et al.^15^ described that five of 13 cases needed additional surgery after initial socket reconstruction. Using the conformer however, a hard palate graft is not necessary. Standard, non-custom-made conformers are available as a reasonable alternative, but do not offer the advantages of optimal size, the ability to adapt the fixation holes exactly where needed, the possibility to fixate upper and lower eyelids to the conformer so that fornices are well formed, and the easy transformation to the cosmetic ocular prosthesis which is based on the 3D-printed conformer. Obviously, the costs for producing 3D-printed objects depend on access to a 3D-printer which is by far the most expensive part of the process. If such a printer is available in the clinic, or the ocularist comes to an agreement with a company to share a printer, it is also possible to add the conformers to an already planned print session intended for other objects. In this way, the costs are limited to the design time, and the print material. The resin we used is about €257 a package for 1000 ml, and as the average conformer is 0.8 ml we can theoretically print 1250 conformers out of one package, leading to an average cost of 20 cents per conformer. If a printer needs to be purchased solely for the purpose of designing these conformers the total costs might not outweigh the benefits.

To avoid disturbing sufficient tissue, three cases were initially only treated for one fornix because there was a moderate opposite fornix. We noted the socket reshaped after the procedure, ultimately needing a second surgery also on this opposite fornix. This made us adapt the procedure to preferably one large graft that reconstructs both upper and lower fornices, enabling a better formation and fixation of the complete socket. It has to be noted that for this procedure (so preferably addressing both fornices), the harvested buccal mucosal grafts should be as large as possible. The size of this graft can be enlarged by perforating holes and stretching the graft, like with skin transplants. Additionally, as the mucosa will re-epithelialize intraorally, it is possible to repeat this procedure multiple times. Alternatives for buccal mucosa can be nasal mucosa, amniotic membrane with or without mitomycin, and synthetic options such as polymers or collagens serving as scaffolds for self-regenerating progenitor conjunctival epithelial and goblet cells.^16^ It should be evaluated whether these scaffolds will be able to result in viable mucosal tissue in recurrent contracted cases, since it may need a certain amount of healthy pre-operative mucosal tissue in the recipient.

A strength of our study is that it presents a relatively simple innovative way to a complex problem: to fixate a mucosal graft using a custom-made conformer, which can be worn during a relatively long period and ultimately benefits the socket lining. In all but one case our treatment goal was reached, at least for the relatively long follow-up time of 6–36 months. A limitation is the small number of cases. Another limitation is that we did not repair the (usually previously existing) entropion using this method. Our primary goal in our series was the retainment of a prosthesis. Although not all patients wished for additional improvement, we might consider correcting entropion as a secondary procedure or perhaps adjust the surgery to a one-step procedure including a lid-split procedure, or the use of a palatum component during the primary surgery.

In conclusion, we described nine cases for whom custom-made, 3D-printed conformers have aided fixation of mucosal transplants in contracted sockets where previous surgeries had failed, enabling the retention of a well-fitted ocular prosthesis in eight cases. We advise initial treatment of both upper and lower fornices to avoid subsequent surgeries. In some cases, extra lid surgery is desirable for cosmetic, but not essential for functional reasons.
